# The associations of radiological features of high-resolution computed tomography with the outcomes of transbronchial cryobiopsy in interstitial lung diseases: A cohort study

**DOI:** 10.3389/fmed.2022.959129

**Published:** 2022-08-02

**Authors:** Guowu Zhou, Yanhong Ren, Jun Li, Ting Yang, Nan Su, Ling Zhao, Shumeng Wang, Dan Wang, Ying Li, Zheng Tian, Ruihong Liu, Huaping Dai, Chen Wang

**Affiliations:** ^1^Department of Pulmonary and Critical Care Medicine, Center of Respiratory Medicine, China-Japan Friendship Hospital, National Center for Respiratory Medicine, Institute of Respiratory Medicine, Chinese Academy of Medical Sciences, National Clinical Research Center for Respiratory Diseases, Beijing, China; ^2^Department of Pathology, China-Japan Friendship Hospital, Beijing, China; ^3^Chinese Academy of Medical Sciences, Peking Union Medical College, Beijing, China

**Keywords:** cryobiopsy, interstitial lung diseases, HRCT, complications, diagnostic yields

## Abstract

**Background:**

Transbronchial cryobiopsy (TBCB) is a critical procedure in the diagnosis of interstitial lung diseases (ILD). The associations between high-resolution computed tomography (HRCT) features and outcomes of TBCB were unknown.

**Methods:**

This study was conducted as a single-center prospective cohort study between September 2018 and January 2020 (NCT04047667). HRCT was obtained before performing TBCB. The clinical and radiological characteristics, complications, pathological and multidisciplinary discussion (MDD) diagnoses were recorded. The relationships between HRCT features and outcomes of TBCB were analyzed.

**Results:**

TBCB was performed on 216 ILD patients. The radiological features usually interstitial pneumonia (UIP) or probable UIP, indeterminate for UIP, ground-glass opacities (GGO) and cysts were found in 55 (25.5%), 38 (17.6%), 84 (38.9%) and 9 (4.2%) patients, respectively. And 118 (54.6%) patients had high HRCT score (involved lung proportion ≥50%) in the biopsied lobe. Multivariate analysis suggested radiological probable UIP pattern may be an independent risk factor for moderate bleeding (OR = 4.304; 95% CI: 1.383–13.393; *P* = 0.012), while GGO may be a protective factor from moderate bleeding (OR = 0.173, 95% CI: 0.043–0.687; *P* = 0.013). The pathological diagnostic yield in patients presenting cysts on HRCT was significantly lower than others (44.4 vs. 87.9%; *P* = 0.009). While performing TBCB in the lobe with high HRCT score increased pathological diagnostic yield (91.5 vs. 79.6%; *P* = 0.022). Neither pneumothorax nor MDD diagnostic yields were found to be associated with HRCT features.

**Conclusions:**

HRCT features were associated with moderate bleeding and pathological diagnosis. Pre-TBCB assessments of HRCT pattern and scores were helpful for bronchoscopists to make a better patient selection and procedure planning.

## Highlights

- HRCT features were associated with transbronchial cryobiopsy-related moderate bleeding and pathological diagnosis.
- Pre-procedure assessments of HRCT features were helpful for bronchoscopists to make a better patient selection and procedure planning.

## Introduction

Transbronchial cryobiopsy (TBCB) is a critical procedure in the assessment of patients with suspected interstitial lung diseases (ILD) when an accurate diagnosis cannot be made solely based on clinical and radiological assessments. Recent reports have suggested that the diagnostic value of TBCB approaches that of surgical lung biopsy (SLB) (1–4). However, the complication rates (such as pneumothorax and significant bleeding) and diagnostic yields reported by different investigations varied. Pneumothorax rate varied from 1.9 to 19.2%, moderate to severe bleeding rate varied from 4.0 to 56.4%, histopathological diagnostic yield varied from 40.0 to 95.1% (5, 6). Several procedural factors have been thought to be associated with the safety profile and diagnostic efficacy of TBCB, such as the cryoprobe-pleura distance, cryoprobe type, guidance method, and the number of cryobiopsies (6–8). However, there are few studies investigating the associations between HRCT features and the outcomes of TBCB. Ravaglia's study found pneumothorax was much more frequent in patients with evaluated grading of the distribution of reticular abnormalities, traction bronchiectasis and honeycombing on HRCT, but no other correlation was found (6). In our preliminary studies, we found that the complication rates were significantly associated with high-resolution computed tomography (HRCT) pattern but not with cryoprobe type (9, 10). The risk of moderate-to-severe bleeding was obviously higher in patients with fibrotic HRCT pattern than those with non-fibrotic pattern.

In order to furtherly demonstrate the potential value of HRCT features on predicting the outcomes of TBCB, we analyze it based on a prospective cohort study in which cryoprobe placement was guided by three dimensional (3D) images acquired by cone beam computed tomography (CBCT) during TBCB. We present the following article in accordance with the STROBE reporting checklist.

## Methods

### Patients

This study was conducted as an updated single-center prospective cohort study concerning on the CBCT guided TBCB for ILD. The trial was conducted in accordance with the Declaration of Helsinki and the Harmonized Tripartite Guideline for Good Clinical Practice from the International Conference on Harmonization. This study was reviewed and approved by the Ethics Committee of China-Japan Friendship Hospital and was registered at clinicaltrial.gov (NCT04047667). Written informed consent was obtained from all patients enrolled. Participant registration was carried out between September 2018 to January 2020.

**Figure 1:**
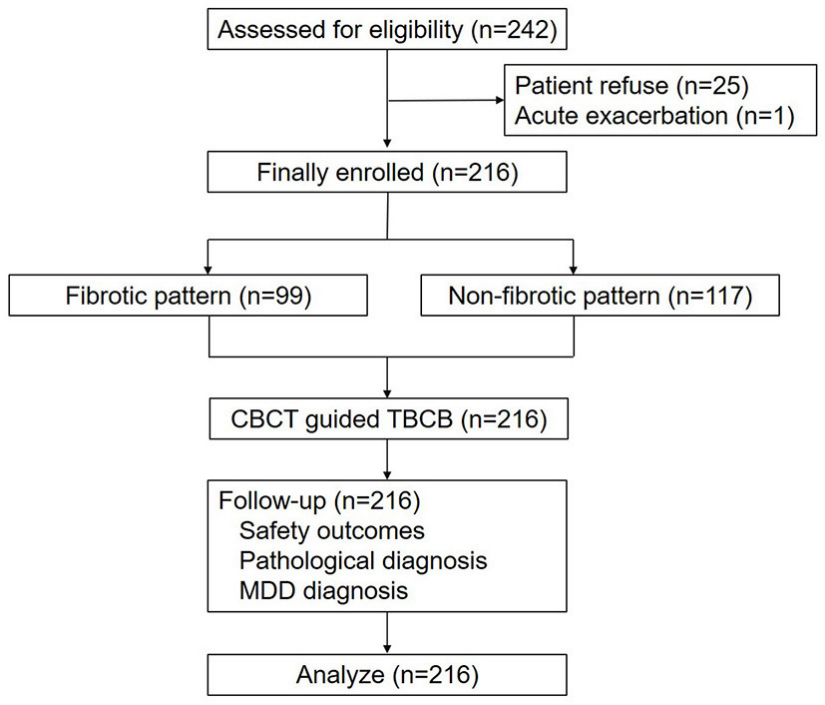
Flow diagram of the enrolment and follow-up processes for the patients who underwent transbronchial cryobiopsy (TBCB).

All patients diagnosed with ILD who met the following eligibility criteria were recommended to undergo TBCB under guidance by CBCT (Figure 1): aged > 18 years old with evidence of diffuse parenchymal lung disease; a diagnosis of ILD that could not be established after integration of clinical data, laboratory tests, and HRCT features; forced vital capacity (FVC) > 50%; and diffusing capacity of the lung for CO (DLCO) > 35%. Patients who met any of the following criteria were excluded from this study: acute exacerbation in the previous 30 days; bleeding diathesis; anticoagulant therapy; current use of antiplatelet drugs; pulmonary hypertension; respiratory failure; liver or kidney dysfunction; cardiac insufficiency; or platelet count < 50 × 10^9^/L. Eventually, 216 patients were enrolled.

### HRCT protocol and assessment

CT scans were obtained using a second-generation, dual-source CT system (SOMATOM Definition Flash, Siemens Healthineers, Forchheim, Germany) with the following parameters before biopsy: 120 kV reference tube voltage, 110 quality reference mAs tube current with automatic exposure control (CarekV,CareDose4D), and 0.5 seconds scanning time, and 128×0.6-mm detector configuration, pitch 1.2. Images were reconstructed with slice thickness of 1mm, and reconstruction increment of 1.0 mm. Images were displayed using lung window [width: 1,600 Hounsfield units (HU); level: −600 HU] and mediastinal window settings (width: 350 HU; level: 50 HU). No contrast was administered. Two experienced physicians (G. Zhou and Y. Ren) independently reviewed each patient's HRCT features. When disagreements between them occurred, or their results did not accord with the HRCT reports from the department of radiology, they consulted the radiological expert (M. Liu) during multidisciplinary discussions (MDD).

Fibrotic pattern included the following HRCT features (11, 12): honeycombing or reticulation with traction bronchiectasis (UIP or probable UIP), reticulation predominant (indeterminate for UIP), and ground-glass opacities with traction bronchiectasis or honeycombing (others).

Non-fibrotic pattern included the following HRCT features: ground-glass opacities predominant without traction bronchiectasis or honeycombing, military nodules, consolidation, cystic manifestation, and others such as mosaic attenuation.

HRCT scores were assessed according to the protocol described by Kazerooni et al. (13). Both observers initially scored the limited CT images, with the three images taken at the level of the aortic arch, the carina, and 1 cm above the diaphragm and later scored the CT images of the entire lungs. Each lobe of the lung was scored on a scale of 0–5 depending on the percentage of each lobe involved: 0, no lung involved; 1, manifestations involving ≤5% of the lobe; 2, manifestations involving 5–25% of the lobe; 3, manifestations involving 25–49%; 4, manifestations involving 50–75% of the lobe; 5, manifestations involving >75% of the lobe. The scores for each lobe were averaged for both readers for data analysis. The score of the entire lungs were averaged for all five lobes. Scale more than 3 was defined as high HRCT score.

### TBCB procedure

TBCB was conducted following the protocol of procedure as previously described (9, 10). Routine post-procedure CBCT or X-ray imaging was used to screen for acute pneumothorax. Bleeding severity was graded according to the following scale (14): no bleeding (traces of blood not requiring suctioning), mild bleeding (requiring suction to clear but no other endoscopic procedures), moderate bleeding (requiring endoscopic procedures such as bronchial occlusion-collapse and/or instillation of ice-cold saline), and severe bleeding (causing hemodynamic or respiratory instability, requiring tamponade or other surgical interventions, transfusions, or admission to the intensive care unit).

### Follow-up

For each patient, electrocardiogram, blood pressure, and oxygen saturation were monitored for 24 h after bronchoscopy. Chest X-rays were performed for patients who exhibited discomfort or disease progression without any other reason.

Histopathological diagnosis was conducted by an experienced pathologist. Multidisciplinary discussions (MDD) based on clinical presentations, radiological findings, and TBCB histopathological features were conducted for each patient. All patients underwent a 30-day follow-up for intensive care unit admission, disease progression, and death after TBCB.

### Endpoints and sample size

The primary endpoints were defined as the incidences of pneumothorax and moderate-severe bleeding. The second endpoints included diagnostic yields, specimen quality, cryoprobe re-position rates after CBCT guidance, and procedure duration. The estimated sample size was designed to have ~80% power for detecting a 6% decrease in the pneumothorax rate (from 9 to 3%), or a 10% decrease in the moderate-severe bleeding rate (from 20 to 10%) after CBCT guidance.

### Statistical analysis

All patients competed 30-day follow-up and were included in this analysis. Anthropometric and lung function data were expressed as mean ± SD. All remaining results were presented as descriptive statistics, absolute numbers, and percentages. Continuous data were tested for normality using the Shapiro-Wilk test. Differences in complication rates were analyzed for statistical significance using the Fisher exact test. Multivariate logistic regression analyses were used to analyze the associations between safety outcomes and HRCT features, as well as between diagnostic yields and HRCT features. Statistical analyses were performed using STATA software (version 14, StataCorp, College Station, TX, USA), and a *p* value < 0.05 was considered statistically significant.

## Results

### Patients characteristics

Of 242 patients eligible for enrolment in the study, 26 were excluded due to patient refusal (*n* = 25) or acute exacerbation (*n* = 1) prior to the procedure. Consequently, 216 patients (male-to-female ratio 1.4, 126/90) were enrolled and underwent TBCB (Figure 1), with a mean age of 55.2 ± 11.8 years; FVC was 89.4 ± 21.2% and DLCO was 67.7 ± 18.5% (Table 1). Fibrotic pattern on HRCT were found in 99 (45.8%) patients: reticulation predominant in 38 (17.6%) patients; honeycombing or reticulation with traction bronchiectasis in 55 (25.5%) patients; GGO with traction bronchiectasis or honeycombing in 6 (2.8%) patients. Non-fibrotic pattern on HRCT were found in 117 (54.2%) patients: GGO predominant in 84 (38.9%) patients; military nodules in 11 (5.1%) patients; consolidation in 11 (5.1%) patients; cystic lesion in 9 (4.2%) patients and others in 2 (0.9%) patients. A total of 80 (37.0%) patients had HRCT scores of more than 3 for all lobes of the lung, while 118 (54.6%) patients had HRCT scores of more than 3 for the biopsied lobe. The risk of moderate bleeding (no severe bleeding occurred) and pneumothorax were 9.7% (21/216) and 2.8% (6/216), respectively. The pathological and MDD diagnostic yields were 86.1% (186/216) and 91.2% (197/216).

**Table 1:** Clinical characteristics in interstitial lung diseases (ILD) patients undergoing transbronchial cryobiopsy (TBCB).

**Patients**	***N* = 216**
Median age (SD), year-old	55.2 (11.8)
Male-to-female ratio	126/90 (1.4)
Smokers	101 (46.8%)
Environmental or occupational history	67 (31.0%)
Mean FVC (SD), %pred	89.4 (21.2)
Mean DLCO (SD), %pred	67.7 (18.5)
Fibrotic pattern	99 (45.8%)
UIP or Probable UIP pattern	55 (25.5%)
Indeterminate for UIP pattern	38 (17.6%)
Other fibrotic pattern	6 (2.8%)
Non-fibrotic pattern	117 (54.2%)
GGO	84 (38.9%)
Profuse micronodules	11 (5.1%)
Consolidation	11 (5.1%)
Cysts	9 (4.2%)
Others	2 (0.9%)
HRCT score	
≤ 3	136 (63.0%)
>3	80 (37.0%)
HRCT score of the target lobe	
≤ 3	98 (45.4%)
>3	118 (54.6%)
Cryoprobe size	
1.9 mm probe	52 (24.1%)
2.4 mm probe	164 (75.9%)
Biopsy site	
Single segment	79 (36.6%)
Multiple segments	137 (63.4%)
Number of specimen (SD)	3.5 (1.0)
Procedure duration (SD), min	37.4 (13.9)
Outcomes	
Moderate-to-severe bleeding	21 (9.7%)
Pneumothorax	6 (2.8%)
Pathological diagnosis	186 (86.1%)
MDD diagnosis	197 (91.2%)

*SD, standard deviation; FEV1, forced expiratory volume in 1 s; FVC, forced vital capacity; DLCO, diffusing capacity of the lung for CO; HRCT, high-resolution computed tomography; CBCT, cone beam computed tomography; RET, reticulation; TB, traction bronchiectasis; HC, honeycombing; GGO, ground-glass opacities*.

Multivariate analysis (Table 2) indicated that FVC % predictive less than 70%, number of specimen <3, and HRCT fibrotic pattern were associated with increased risk of moderate bleeding while environmental history was associated with decreased risk of moderate bleeding. Age more 65 year-old was associated with increased risk of pneumothorax. Gender of male was associated with lower MDD diagnostic yields while smoking status was associated with higher MDD diagnostic yields.

**Table 2:** Multivariate analysis for the associations between clinical characteristics and the outcomes of transbronchial cryobiopsy in ILD patients.

**Characteristics**	**Moderate bleeding**		**Pneumothorax**		**Pathological diagnosis**		**MDD diagnosis**	
	**OR (95% CI)**	***P***	**OR (95% CI)**	***P***	**OR (95% CI)**	***P***	**OR (95% CI)**	***P***
Age > 65 year-old	1.102 (0.321, 3.790)	0.877	**6.973 (1.080, 45.029)**	**0.041**	0.780 (0.258, 2.361)	0.661	0.322 (0.064, 1.624)	0.170
Male	0.580 (0.107, 3.147)	0.527	0.356 (0.013, 10.077)	0.545	0	0.991	**0.041 (0.002, 0.792)**	**0.035**
Smoker	1.425 (0.253, 8.041)	0.688	3.368 (0.138, 82.333)	0.457	0	0.991	**30.788 (1.567, 604.767)**	**0.024**
Environmental or occupational history	**0.159 (0.031, 0.821)**	**0.028**	1.323 (0.173, 10.141)	0.788	0.654 (0.233, 1.835)	0.420	0.625 (0.191, 2.052)	0.439
FVC %pred <70%	**6.156 (1.407, 26.935)**	**0.016**	1.169 (0.086, 15.923)	0.907	0.342 (0.067, 1.749)	0.198	2.079 (0.565, 7.645)	0.271
DLCO %pred <50%	0.153 (0.018, 1.333)	0.089	2.314 (0.215, 24.754)	0.488	1.292 (0.285, 5.857)	0.740	1.605 (0.359, 7.184)	0.536
2.4-mm cryoprobe	0.631 (0.170, 2.335)	0.490	N/A		2.098 (0.616, 7.144)	0.236	1.097 (0.303, 3.970)	0.888
Multiple biopsy sites	0.679 (0.191, 2.410)	0.549	0.985 (0.124, 7.787)	0.988	0.896 (0.318, 2.526)	0.835	0.950 (0.306, 2.954)	0.930
Number of specimen <3	**2.681 (1.154, 6.232)**	**0.022**	0.697 (0.158, 3.073)	0.634	0.745 (0.360, 1.543)	0.429	0.652 (0.277, 1.535)	0.328
Fibrotic pattern	**8.660 (2.515, 29.821)**	**0.001**	0.790 (0.113, 5.540)	0.812	0.703 (0.263, 1.876)	0.481	0.769 (0.250, 2.364)	0.647

*MDD, multidisciplinary discussion*.

*Bold values indicate significant p-value < 0.05*.

### HRCT features and safety profile

In general, the risk of moderate bleeding in patients with HRCT fibrotic patterns was significantly higher than those with HRCT non-fibrotic patterns (15.2 vs. 5.1%, *P* = 0.013; Table 3). The moderate bleeding rates in the subgroup of UIP or probable UIP pattern, indeterminate for UIP pattern and other fibrotic patterns were 14.5, 15.8, and 16.7% respectively. The moderate bleeding rate in the patients with GGO was 4.8%, which was significantly lower than others (*P* = 0.05). In two out of nine patients with cysts on HRCT occurred moderate bleeding (22.2%, *P* = 0.214). Multivariate analysis suggested that honeycombing or reticulation with bronchiectasis on HRCT may be an independent risk factor for moderate bleeding (OR = 4.304; 95% CI: 1.383–13.393; *P* = 0.012; Table 4), while GGO predominant on HRCT may be a protective factor from moderate bleeding (OR = 0.173, 95% CI: 0.043–0.687; *P* = 0.013; Table 4). No relationship between HRCT score and moderate bleeding was found.

**Table 3:** The safety profile and diagnostic efficacy of transbronchial cryobiopsy (TBCB) in patients with different HRCT features.

**Characteristics**	**Moderate bleeding**		**Pneumothorax**		**Pathological diagnosis**		**MDD diagnosis**	
	***N* (%)**	***P***	***N* (%)**	***P***	***N* (%)**	***P***	***N* (%)**	***P***
**HRCT manifestations**								
Fibrotic pattern (*n* = 99)	15 (15.2%)	**0.013**	3 (3.0%)	0.835	86 (86.7%)	0.767	90 (90.9%)	0.888
UIP or Probable UIP (*n* = 55)	8 (14.5%)	0.162	2 (3.6%)	0.979	45 (81.8%)	0.286	51 (92.7%)	0.852
Indeterminate for UIP (*n* = 38)	6 (15.8%)	0.164	0 (0.0%)	0.546	35 (92.1%)	0.239	33 (86.8%)	0.296
Other fibrotic pattern (*n* = 6)	1 (16.7%)	0.463	1 (16.7%)	0.157	6 (100.0%)	1.000	6 (100.0%)	1.000
Non-fibrotic pattern (*n* = 117)	6 (5.1%)		3 (2.6%)		100 (85.5%)		107 (91.5%)	
GGO (*n* = 84)	4 (4.8%)	**0.050**	3 (3.6%)	0.887	75 (89.3%)	0.282	78 (92.9%)	0.494
Profuse micronodules (*n* = 11)	0 (0.0%)	1.000	0 (0.0%)	1.000	10 (90.9%)	1.000	9 (81.8%)	0.250
Consolidation (*n* = 11)	0 (0.0%)	1.000	0 (0.0%)	1.000	10 (90.9%)	1.000	11 (100.0%)	1.000
Cysts (*n* = 9)	2 (22.2%)	0.214	0 (0.0%)	1.000	4 (44.4%)	**0.003**	7 (77.8%)	0.182
Others (*n* = 2)	0 (0.0%)	1.000	0 (0.0%)	1.000	1 (50.0%)	0.259	2 (100.0%)	1.000
HRCT score in whole		0.072		0.812		0.803		0.606
≤ 3 (*n* = 136)	17 (12.5%)		3 (2.2%)		116 (85.3%)		123 (90.4%)	
>3 (*n* = 80)	4 (5.0%)		3 (3.8%)		70 (87.5%)		74 (92.5%)	
HRCT score of target lobe		0.254		0.309		**0.012**		0.251
≤ 3 (*n* = 98)	12 (12.2%)		1 (1.0%)		78 (79.6%)		87 (88.8%)	
>3 (*n* = 118)	9 (7.6%)		5 (4.2%)		108 (91.5%)		110 (93.2%)	

*HRCT, high-resolution computed tomography; MDD, multidisciplinary discussion. RET, reticulation; TB, traction bronchiectasis; HC, honeycombing; GGO, ground-glass opacities*.

*Bold values indicate significant p-value < 0.05*.

**Table 4:** Multivariate analysis for the associations between HRCT features and the outcomes of transbronchial cryobiopsy in ILD patients.

**Characteristics**	**Moderate bleeding**		**Pneumothorax**		**Pathological diagnosis**		**MDD diagnosis**	
	**OR (95% CI)**	***P***	**OR (95% CI)**	***P***	**OR (95% CI)**	***P***	**OR (95% CI)**	***P***
Fibrotic pattern	**8.660 (2.515, 29.821)**	**0.001**	0.790 (0.113, 5.540)	0.812	0.703 (0.263, 1.876)	0.481	0.769 (0.250, 2.364)	0.647
UIP or probable UIP	**4.304 (1.383, 13.393)**	**0.012**	1.954 (0.274, 13.950)	0.504	0.450 (0.165, 1.227)	0.119	1.277 (0.345, 4.735)	0.714
Indeterminate for UIP	2.675 (0.787, 9.089)	0.115	N/A		1.649 (0.408, 6.655)	0.482	0.392 (0.108, 1.419)	0.153
Other fibrotic pattern	2.465 (0.181, 33.595)	0.498	5.906 (0.219, 159.522)	0.291	N/A		N/A	
Non-fibrotic pattern								
GGO	**0.173 (0.043, 0.687)**	**0.013**	2.108 (0.308, 14.444)	0.448	0.424 (0.152, 1.182)	0.101	0.645 (0.211, 1.972)	0.442
Profuse micronodules	N/A		N/A		1.530 (0.166, 14.080)	0707	0.257 (0.040, 1.659)	0.153
Consolidation	N/A		N/A		2.489 (0.258, 24.060)	0.431	N/A	
Cysts	2.878 (0.462, 17.918)	0.257	N/A		**0.111 (0.022, 0.573)**	**0.009**	0.376 (0.054, 2.639)	0.325
Others	N/A		N/A		0.221 (0.010, 4.743)	0.335	N/A	
HRCT score in whole	0.208 (0.042, 1.032)	0.055	0.443 (0.053, 3.704)	0.453	0.273 (0.046, 1.619)	0.153	1.177 (0.234, 5.933)	0.843
HRCT score of target lobe	1.000 (0.277, 3.612)	1.000	5.904 (0.354, 98.577)	0.216	**7.172 (1.335, 38.532)**	**0.022**	1.751 (0.406, 7.543)	0.452

*HRCT, high-resolution computed tomography; MDD, multidisciplinary discussion. RET, reticulation; TB, traction bronchiectasis; HC, honeycombing; GGO, ground-glass opacities*.

*Bold values indicate significant p-value < 0.05*.

The risk of pneumothorax had no significant differences between fibrotic and non-fibrotic patterns (3.0 vs. 2.6%). There was a trend for the increased incidence of pneumothorax in the patients who underwent TBCB in the lobe with high HRCT score, but without no significant statistical difference (4.2 vs. 1.0%, *P* = 0.309). The associations between HRCT features and pneumothorax were not found by multivariate analysis in this study (Tables 3, 4).

### HRCT features and diagnostic yields

The pathological diagnostic yield in the patients presenting cysts on HRCT was significantly lower than others (44.4 vs. 87.9%, *P* = 0.003; Table 3). When TBCB was performed in the lobe with high HRCT score, the pathological diagnostic yield would significantly increase (91.5 vs. 79.6%, *P* = 0.012; Table 3). Multivariate analysis also demonstrated that cysts on HRCT predicted failure of pathological diagnosis (OR = 0.111, 95% CI: 0.022–0.573; *P* = 0.009; Table 4), while performing TBCB in the lobe with high HRCT score may improve pathological diagnostic yields (OR = 7.172, 95% CI: 1.335–38.532; *P* = 0.022; Table 4).

The associations between HRCT features and MDD diagnostic yields were not found in this study (Tables 3, 4).

## Discussion

This is the first prospective study to investigate the association between HRCT features and the outcomes of TBCB in ILD patients. Our results offer new insights into the relationship between radiological manifestations and TBCB outcomes, it may be helpful for bronchoscopists to make a better patient selection in order to improve procedure safety and diagnostic yields. Prophylactical placement of bronchial blocker should be routinely used if high risk of significant bleeding is suspected and TBCB should be conducted in the lobe with high HRCT score.

Bleeding and pneumothorax are the most frequent complications of TBCB (15, 16). Previous studies suggested the complications were related with the cryoprobe-to-pleura distance and procedure methods (7, 8). Casoni et al. (17) found that the risk of pneumothorax was increased when fragments of pleura were present in the biopsy. Ravaglia et al. indicated pneumothorax was much more frequent in patients who underwent TBCB by using 2.4-mm probe (6). However, in our study TBCB was conducted under the guidance of 3D CBCT images which were reviewed in axial, coronal, and sagittal planes to accurately assess the cryoprobe-to-pleura distance. Few patients presented fragments of pleura and the pneumothorax rate was much lower than the average incidence by pooled previously reported studies in which TBCB were mostly performed under fluoroscopic guidance (2.8 vs. 9.4%). The varied risks of pneumothorax may be due to the difference of guiding accuracies between 3D CBCT and traditional fluoroscopy. The relationships between safety profile and HRCT features were analyzed in our study without the influence of guiding accuracy, and the results may be more convincible. Our study did not found any associations between pneumothorax and HRCT features, besides procedure methods. On the other hand, we found the risk of moderate bleeding could be predicted by pre-procedure HRCT pattern: radiological UIP or probable UIP pattern was associated with higher risk of moderate bleeding while GGO was associated with lower risk.

As we known, surgical lung biopsy (SLB) was not recommended in the patients having HRCT images presenting a typical UIP pattern, because of the relatively high complication rates and mortality in this part of patients (12). Compared to SLB, TBCB had lower morbidity and it was suggested that sometimes TBCB could be proposed in patients with a typical radiological UIP pattern (7). In our study, we found honeycombing or reticulation with bronchiectasis manifestations on HRCT, which was the typical radiological abnormality of UIP or probable UIP pattern, was significantly associated with TBCB related moderate bleeding (OR = 4.304, 95% CI: 1.383–13.393). Furthermore, Ravaglia et al. found that pneumothorax was much more frequent in patients with evaluated grading of the distribution of reticulation, traction bronchiectasis and honeycombing (6). As a result, TBCB should be performed with all the proper precautions and prophylactical placement of bronchial blocker should be routinely used in this kind of patients. As for the typical radiological UIP pattern, which presented obvious honeycombing with or without bronchiectasis manifestations, broadening indication of TBCB to this part of patients should be carefully assessed by investigations.

For the patients with cystic manifestation on HRCT, transbronchial lung biopsy was one of the valuable methods for pathological diagnosis. Previous study reported that Langerhans cell histiocytosis (LCH) and lymphangioleiomyomatosis (LAM) could be successfully diagnosed by TBCB (18–20). However, our study showed the pathological diagnostic yield for the patients with cysts on HRCT was low (44.4%, *P* = 0.003). And multivariate analysis demonstrated cystic manifestation predicted failure of pathological diagnosis. The low diagnostic yield may be explained by the small scale of lesion. The HRCT score of cysts in the biopsied lobe of lung was less than 3 in most patients [8/9]. Our study suggested the pathological diagnostic yield in patients with HRCT score in the target lobe less than 3 was significantly lower than those patients with HRCT score more than 3. On the other hand, our study showed a relatively high incidence of TBCB related moderate bleeding (2/9, 22.2%). As a result, the safety and diagnostic value of TBCB for cystic manifestation needed to be proved by more investigations with larger sample size.

## Conclusions

The HRCT features were associated with moderate bleeding and pathological diagnostic yields. Radiological UIP or probable UIP pattern was associated with higher risk of moderate bleeding while GGO was associated with low risk. Cystic manifestation predicted failure of pathological diagnosis while performing TBCB in the lobe with high HRCT score may increase pathological diagnosis. Pre-procedure assessment of HRCT pattern was helpful for bronchoscopists to make a better patient selection and procedure planning, in order to improve procedure safety and diagnostic yields.

## Data availability statement

The raw data supporting the conclusions of this article will be made available by the authors, without undue reservation.

## Ethics statement

The studies involving human participants were reviewed and approved by Ethics Committee of China-Japan Friendship Hospital. The patients/participants provided their written informed consent to participate in this study.

## Author contributions

Conception and design: HD, GZ, YR, and CW. Administrative: HD. Provision of study materials or patients: GZ, YR, JL, TY, NS, LZ, SW, DW, YL, ZT, and RL. Collection and assembly of data, data analysis and interpretation, and manuscript writing: GZ and YR. Revision of manuscript and final approval of manuscript: all authors. All authors contributed to the article and approved the submitted version.

## Funding

This work was supported by Chinese Academy of Medical Sciences Innovation Fund for Medical Sciences (CIFMS, No. 2018-I2M-1-001 to HD), National Key Technologies R&D Program Precision Medicine Research (No. 2016YFC0901101 to HD).

## Conflict of interest

The authors declare that the research was conducted in the absence of any commercial or financial relationships that could be construed as a potential conflict of interest.

## Publisher's note

All claims expressed in this article are solely those of the authors and do not necessarily represent those of their affiliated organizations, or those of the publisher, the editors and the reviewers. Any product that may be evaluated in this article, or claim that may be made by its manufacturer, is not guaranteed or endorsed by the publisher.
